# Genome-Wide Mapping of Bivalent Histone Modifications in Hepatic Stem/Progenitor Cells

**DOI:** 10.1155/2019/9789240

**Published:** 2019-04-01

**Authors:** Kengo Kanayama, Tetsuhiro Chiba, Motohiko Oshima, Hiroaki Kanzaki, Shuhei Koide, Atsunori Saraya, Satoru Miyagi, Naoya Mimura, Yuko Kusakabe, Tomoko Saito, Sadahisa Ogasawara, Eiichiro Suzuki, Yoshihiko Ooka, Hitoshi Maruyama, Atsushi Iwama, Naoya Kato

**Affiliations:** ^1^Department of Gastroenterology, Graduate School of Medicine, Chiba University, Chiba, Japan; ^2^Department of Cellular and Molecular Medicine, Graduate School of Medicine, Chiba University, Chiba, Japan; ^3^Department of Laboratory Medicine, Lund University, Lund, Sweden; ^4^Department of Transfusion Medicine and Cell Therapy, Chiba University Hospital, Chiba, Japan

## Abstract

The “bivalent domain,” a distinctive histone modification signature, is characterized by repressive trimethylation of histone H3 at lysine 27 (H3K27me3) and active trimethylation of histone H3 at lysine 4 (H3K4me3) marks. Maintenance and dynamic resolution of these histone marks play important roles in regulating differentiation processes in various stem cell systems. However, little is known regarding their roles in hepatic stem/progenitor cells. In the present study, we conducted the chromatin immunoprecipitation (ChIP) assay followed by high-throughput DNA sequencing (ChIP-seq) analyses in purified delta-like 1 protein (Dlk^+^) hepatic stem/progenitor cells and successfully identified 562 genes exhibiting bivalent domains within 2 kb of the transcription start site. Gene ontology analysis revealed that these genes were enriched in developmental functions and differentiation processes. Microarray analyses indicated that many of these genes exhibited derepression after differentiation toward hepatocyte and cholangiocyte lineages. Among these, 72 genes, including *Cdkn2a* and *Sox4*, were significantly upregulated after differentiation toward hepatocyte or cholangiocyte lineages. Knockdown of *Sox4* in Dlk^+^ cells suppressed colony propagation and resulted in increased numbers of albumin^+^/cytokeratin 7^+^ progenitor cells in colonies. These findings implicate that derepression of *Sox4* expression is required to induce normal differentiation processes. In conclusion, combined ChIP-seq and microarray analyses successfully identified bivalent genes. Functional analyses of these genes will help elucidate the epigenetic machinery underlying the terminal differentiation of hepatic stem/progenitor cells.

## 1. Introduction

Before 2000, most research on liver development and differentiation was performed using morphological approaches in knockout mice [1]. Thus, many transcription factors with important roles in hepatocyte and cholangiocyte differentiation have been reported [2, 3]. Advances in cell sorting technology since the beginning of the 21st century have led to progress in the isolation and identification of hepatic stem/progenitor cells [4], and it has become possible to analyze signal transduction pathways and molecules involved in the maintenance and/or differentiation of stem cells [5–7].

Epigenetic mechanisms, including DNA methylation and histone modification, are essential for cell fate decisions and differentiation during embryogenesis [8]. Particularly, histone modifications are dynamically regulated by enzymes that add or remove these modifications [9], and these modifications have been described as extremely important in developmental processes in both the liver and pancreas [10]. Although polycomb group (PcG) proteins are responsible for transcription-repressive histone H3 trimethylation at lysine 27 (H3K27me3), trithorax group complexes (TrxG) are associated with transcription-active histone H3 trimethylation at lysine 4 (H3K4me3) [11]. In embryonic stem (ES) and tissue-specific stem cells, the promoter regions of genes that regulate differentiation contain “bivalent domains” with both the H3K27me3 and H3K4me3 [12]. This configuration is believed to allow cell fate determination and differentiation to rapidly begin in any direction in response to intracellular and extracellular signals.

Here, we aimed to elucidate the mechanisms through which histone modifications regulate differentiation from the viewpoint of bivalent domains in normal hepatic stem/progenitor cells. We performed the chromatin immunoprecipitation (ChIP) assay followed by high-throughput DNA sequencing (ChIP-seq) analyses in delta-like 1 protein (Dlk^+^) hepatic stem/progenitor cells using anti-H3K4me3 and anti-H3K27me3 antibodies. Next, we performed microarray analyses using RNA isolated from Dlk^+^ and differentiated cells. A comprehensive analysis of these data allowed for determination of bivalent genes and elucidation of epigenetic regulatory machinery of the differentiation process in hepatic stem/progenitor cells.

## 2. Materials and Methods

### 2.1. Mice

Pregnant C57BL/6 mice were purchased from Japan SLC (Hamamatsu, Japan). They were bred and maintained in accordance with our institutional guidelines for the use of laboratory animals.

### 2.2. Purification and Culture of Dlk^+^ Cells

Dlk^+^ cells were prepared from embryonic day (ED) 14.5 fetal livers, as described previously [13]. Briefly, cells were stained with an anti-Dlk antibody (MBL, Nagoya, Japan) followed by exposure to anti-rat IgG-conjugated magnetic beads (Miltenyi Biotec, Bergisch Gladbach, Germany). Dlk^+^ cells were corrected by passing them through cell separation columns under a magnetic field (Miltenyi Biotec).

### 2.3. Colony Assays and Terminal Differentiation Experiments

Dlk^+^ cells (1 × 10^3^ cells/well) were plated on collagen type IV-coated 6-well plates (Becton Dickinson, Franklin Lakes, NJ) and cultured as described elsewhere [13]. At least three independent experiments were performed for colony assays. To evaluate the ability to differentiate into hepatocytes, Dlk^+^ cells were placed on an Engelbreth-Holm-Swarm (EHS) gel (Becton Dickinson) in the presence of oncostatin M (OSM, R&D Systems, Minneapolis, MN). Similarly, the cells were placed on collagen type I gel (Nitta Gelatin, Osaka, Japan) in the presence of tumor necrosis factor- (TNF-) *α* (PeproTech, Rocky Hill, NJ) to examine their potential to differentiate into cholangiocytes.

### 2.4. Flow Cytometry

To examine the purity of Dlk^+^ cells sorted by magnetic cell separation, the cells were stained with a phycoerythrin-conjugated anti-Dlk antibody (MBL) for 30 min on ice. Labeled cells were resuspended in PBS with 1% fetal bovine serum. Propidium iodide (1 *μ*g/ml) was added for the elimination of dead cells. Cell analysis and sorting were performed using FACSCanto and FACSAria II (BD Biosciences, San Jose, CA).

### 2.5. Immunocytochemistry

Colonies derived from Dlk^+^ cells were stained with rabbit anti-albumin (Alb) (GeneTex, San Antonio, TX) and mouse anti-cytokeratin 7 (CK7) (DakoCytomation, Fort Collins, CO) antibodies followed by Alexa Fluor 555-conjugated goat anti-rabbit IgG (Molecular Probes, Eugene, OR) and Alexa Fluor 488-conjugated goat anti-mouse IgG (Molecular Probes) antibodies, respectively. The absolute number of Alb^+^CK7^+^ bipotent cells in each large colony at day 5 of culture was determined in at least 10 large colonies containing more than 100 cells. To examine cellular apoptosis, cells were stained with an anti-caspase 3 (CASP3; Millipore, Billerica, MA) antibody, followed by incubation with Alexa 555-conjugated IgG (Molecular Probes).

### 2.6. Reverse Transcription Polymerase Chain Reaction (RT-PCR)

RNA was extracted using the RNeasy Mini kit (Qiagen, Valencia, CA), following the manufacturer's protocol. cDNA synthesis was performed using the ThermoScript RT-PCR system (Invitrogen, Frederick, MD) with an oligo-dT primer. Quantitative RT-PCR was performed with an ABI PRISM 7300 Sequence Detection System (Applied Biosystems, Foster City, CA) using the Universal ProbeLibrary System (Roche Diagnostics, Mannheim, Germany) following the manufacturer's protocol. The primer sequences are listed in Supplementary Table S1.

### 2.7. Lentiviral Production and Transduction

Lentiviral vectors (CS-H1-shRNA-EF-1a-EGFP) expressing short hairpin RNA (shRNA) targeting murine *SRY-related high-mobility group* (*HMG*) *box 4* (*Sox4*, target sequence: sh-*Sox4*-1, 5′-ACCAACAACGCGGAGAACACT-3′; sh-*sox4*-2, 5′-GCGACAAGATTCCGTTCATCA-3′) and *luciferase* (*Luc*) were constructed using Gateway LR Clonase systems (Invitrogen, Carlsbad, CA). Recombinant lentiviruses were produced as described previously [14]. Cells were transduced with a lentiviral vector in the presence of protamine sulfate (10 *μ*g/ml; Sigma, St. Louis, MO).

### 2.8. Western Blotting

*Sox4*-knockdown cells were selected via cell sorting for enhanced green fluorescent protein (EGFP) expression. Cells were lysed in RIPA buffer (50 mM Tris (pH 8.0), 150 mM NaCl, 1 mM EDTA (pH 8.0), 1% Triton X-100, 0.1% sodium deoxycholate, and 0.1% SDS) with protease inhibitor cocktail (Roche). Lysates were then sonicated, separated by SDS-PAGE, and transferred to a PVDF membrane. Subsequently, they were subjected to Western blotting using anti-Sox4 (Santa Cruz Biotechnology, Santa Cruz, CA) and anti-tubulin (Oncogene Science, Cambridge, MA) antibodies.

### 2.9. ChIP

ChIP assay was performed as described previously [15]. Briefly, cross-linked chromatin was sonicated into 200–500-base-pair fragments. The immunoprecipitated and input DNA was treated using anti-H3K4me3 (Millipore, Bedford, MA, USA) and anti-H3K27me3 antibodies (Millipore). Normal mouse IgG was used as a negative control. The immunoprecipitated and input DNA was treated with RNase A (Sigma-Aldrich) and proteinase K (Roche) and purified using the QIAquick PCR purification kit (Qiagen). Quantitative PCR was conducted using the ABI Prism 7300 Thermal Cycler with SYBR Premix Ex Taq II (Takara Bio, Otsu, Japan). The primer sequences used are listed in Supplementary Table S2.

### 2.10. ChIP-seq

The reads per million (RPM) mapped read values of the region from 2 kb upstream to 2 kb downstream to the transcription start site (TSS) of the immunoprecipitated samples were divided by the RPM of the corresponding input. The RPM values of the sequenced reads were calculated every 2,000-base-pair bins with a shifting size of 200 base pairs using BEDTools. The RPM values of the sequenced read were calculated using BEDTools. The RPM values of the sequenced reads were calculated using BEDTools and visualized using the Integrative Genomics Viewer (http://www.broadinstitute.org/igv). The ChIP-seq data obtained in this study were deposited in the DNA Data Bank of Japan (DDBJ, accession number DRA006858).

### 2.11. Microarray Analysis

Microarray analysis was conducted using SurePrint G3 Mouse GE microarray (Agilent Technologies, Santa Clara, CA, USA). The microarray was labeled, hybridized, washed, and scanned as described by the manufacturer. The expression value (signal) for each probe set was calculated using GeneSpring GX 12.0 (Agilent). Data were normalized using GeneSpring normalization algorithms (Agilent). Only statistically significant gene expression levels (*P* < 0.05) were recorded as being “detected” above background levels, whereas genes with expression levels lower than this statistical threshold were considered “absent.” To identify differentially expressed genes involved in the differentiation of Dlk^+^ cells, genes exhibiting greater than 2.0-fold changes were selected. Moreover, gene ontology (GO) annotations were performed using the GeneSpring annotation tool (Agilent). The significance of each term was determined using Fisher's exact test and the Bonferroni adjustment for multiple testing. The raw data are available at Gene Expression Omnibus (GEO, accession number: GSE 114833).

### 2.12. Statistical Analysis

Data are presented as mean ± SEM. Statistical differences were analyzed using the Mann–Whitney *U* test. The correlation between histone marks and expression levels was analyzed using Pearson's correlation analysis. *P* values less than 0.05 were considered statistically significant.

## 3. Results

### 3.1. Genome-Wide Analyses of H3K4me3 and H3K27me3 in Hepatic Stem/Progenitor Cells

**Figure 1:**
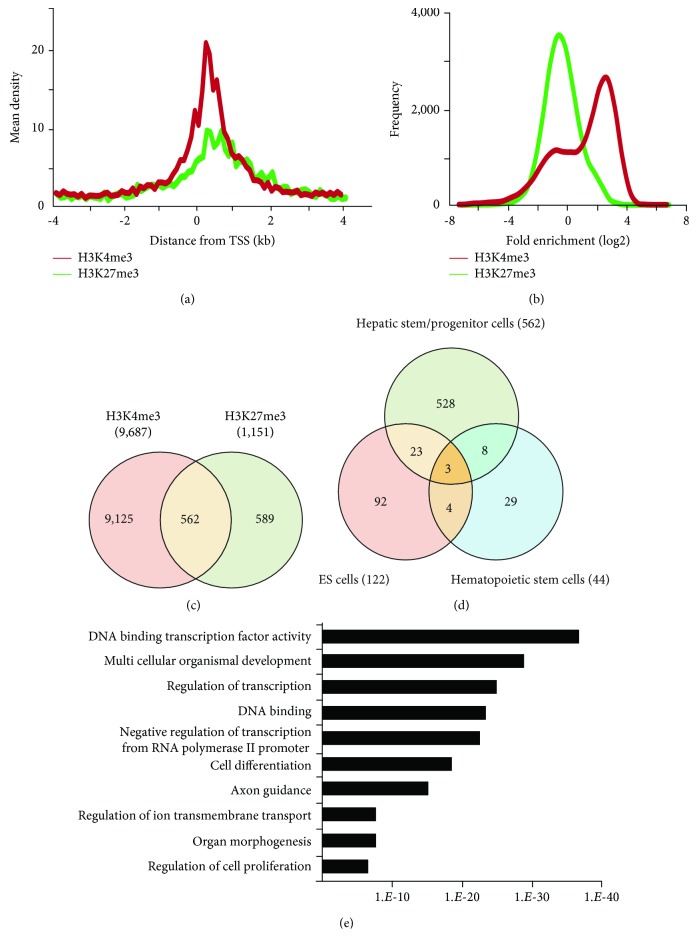
ChIP-seq analyses of freshly isolated Dlk^+^ cells. (a) Distribution of H3K4me3- or H3K27me3-bound probes in the promoter regions (from -2 kb to +2 kb of the TSS). (b) Summary of H3K4me3 or H3K27me3 enrichment detected by ChIP-seq analyses. (c) Venn diagram showing the number of genes with H3K4me3 and/or H3K27me3 modifications with fold enrichment > 2 (log2). (d) Overlap of bivalent genes among the histone modification profiles in ES cells reported by Bernstein et al. [16] and hematopoietic stem cells reported by Weishaupt et al. [17]. (e) GO analyses of genes marked with both H3K4me3 and H3K27me3.

To gain an insight into the histone modification of hepatic stem/progenitor cells, we purified Dlk^+^ cells from fetal livers at ED 14.5 using magnetic-activated cell sorting (MACS). Flow cytometric analysis revealed that the purity of the sorted Dlk^+^ cells was approximately 90% (Supplementary Figure S1). To explore the genome-wide distribution of H3K4me3 and H3K27me3 in hepatic stem/progenitor cells, purified Dlk^+^ cells were subjected to ChIP-seq analysis. Although both the H3K4me3 and H3K27me3 peaks were observed in the slight downstream region of TSS, the H3K27me3 peaks had greater width and centrally depressed signals (Figure 1(a)). We focused on the region within 2.0 kb of the TSSs of the reference sequence genes (Figure 1(b)). Subsequently, ChIP-seq analysis successfully identified 9,687 genes with H3K4me3 enrichment and 1,151 genes with H3K27me3 enrichment greater than 2-fold (log2) of the input levels. In total, 562 genes possessed bivalent chromatin domains containing H3K4me3 and H3K27me3 marks (Figure 1(c)).

It has been reported that polycomb proteins Bmi1 and Ezh2 are required for self-renewal regulation in hepatic stem/progenitor cells as well as in pluripotent stem cells, such as ES cells, and hematopoietic stem cells [5, 13]. These findings suggest that common machinery plays an important role in differentiation regulation in ES cells and somatic stem cells. Therefore, we subsequently compared the list of these genes with the ChIP-seq data examined in mouse ES cells and human/mouse hematopoietic stem cells (HSCs) [16, 17]; 26 and 11 genes with bivalent promoters in hepatic stem/progenitor cells were overlapped with bivalent genes in ES cells and HSCs, respectively (Figure 1(d)). Among them, three genes, namely, *Ebf1*, *Pax6*, and *HoxA1*, were overlapped with genes with bivalent promoters in both ES cells and HSCs. To gain insights into their biological roles, we analyzed the 562 identified bivalent genes. Functional annotations based on GO revealed significant enrichment of genes belonging to the categories “DNA binding,” “transcription,” “development,” and “differentiation” (Figure 1(e)).

### 3.2. Comparison between Histone Marks and Gene Expression Levels

**Figure 2:**
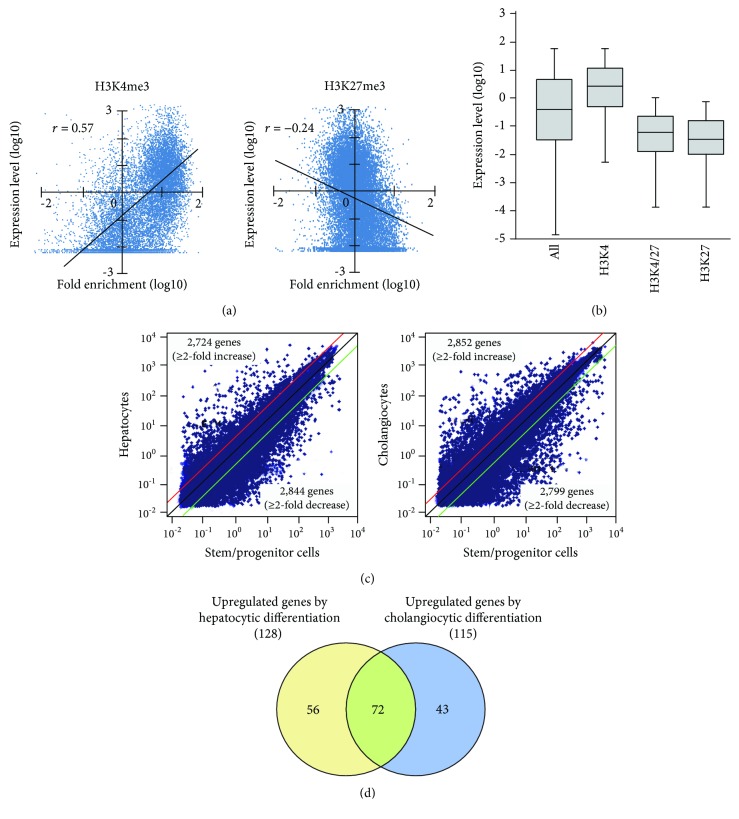
Correlation between histone marks and gene expression level and identification of bivalent genes showing derepression after differentiation induction. (a) Pearson's correlation coefficient analysis revealed that the levels of H3K4me3 enrichment were positively correlated with gene expression levels (*r* = 0.57). By contrast, levels of H3K27me3 enrichment were inversely correlated with gene expression levels (*r* = -0.24). (b) Box plot showing the 25th, 50th, and 75th percentiles of expression for genes with different histone modifications. (c) Scatter diagrams of the microarray analysis. Alterations in gene expression following hepatocytic differentiation in response to OSM and cholangiocytic differentiation in response to TNF-*α* were analyzed via microarray-based expression analysis. The red and green lines represent the borderline for the 2-fold increases and decreases, respectively. (d) Venn diagram depicted the number of bivalent genes showing derepression after differentiation induction.

Initially, based on the microarray data of freshly purified Dlk^+^ cells, we examined the relationship between histone modification patterns and gene expression levels (Figure 2(a)). Pearson's correlation coefficient analysis revealed a positive correlation between gene expression and H3K4me3 levels (*r* = 0.57) and a negative correlation between gene expression and H3K27me3 levels (*r* = −0.24). We compared expression levels among genes marked with H3K4me3, H3K27me3, and both H3K4me3 and H3K27me3 (bivalent) (Figure 2(b)). Genes with H3K4me3 marks showed higher expression levels, whereas those with H3K27me3 marks and bivalent marks (H3K4/27me3) displayed lower expression levels.

### 3.3. Identification of Bivalent Genes Showing Derepression during Differentiation

Next, we cultured Dlk^+^ cells on EHS gel in the presence of OSM to induce differentiation into mature hepatocytes and on collagen type I gel in the presence of TNF-*α* to selectively induce differentiation into cholangiocytes. mRNA extracted from these differentiated cells was subjected to microarray analysis. We successfully identified 2,724 upregulated and 2,844 downregulated genes after the differentiation of Dlk^+^ cells into hepatocytes (Figure 2(c)). Similarly, 2,852 genes were upregulated and 2,799 were downregulated after differentiation into cholangiocytes. Bivalent domains are considered to control the expression of developmental genes, thus allowing timely activation during the differentiation process. To gain insights into epigenetic differentiation regulators, we highlighted the bivalent genes that were derepressed following the differentiation of hepatic stem/progenitor cells. As a result, 128 and 115 genes were upregulated during differentiation into hepatocytes and cholangiocytes, respectively (Figure 2(d)). Among these, 72 genes were commonly upregulated during differentiation toward both the hepatocyte and cholangiocyte lineages (Supplementary Table S3).

### 3.4. Changes of Histone Marks in the *Ink4a*/*Arf* Gene Locus during Differentiation

Among the bivalent genes, the *Ink4a*/*Arf* locus was modified by bivalent histone marks in purified Dlk^+^ cells. *Ink4a*/*Arf* is an important target of PcG, and its transcriptional repression is essential for maintaining self-renewal in hepatic stem/progenitor cells [18]. Consistent with microarray data, real-time PCR analyses demonstrated that remarkable derepression of *Ink4a*/*Arf* was observed after the induction of hepatocytic and cholangiocytic differentiation (Supplementary Figure S2). ChIP analyses revealed high levels of both H3K4me3 and H3K27me3 marks at the *Ink4a*/*Arf* promoter in purified Dlk^+^ cells. However, H3K27me3 levels, but not H3K4me3 levels, were diminished at the locus in mature hepatocytes and cholangiocytes.

### 3.5. Bivalent Histone Modification on the *Sox4* Gene Locus

**Figure 3:**
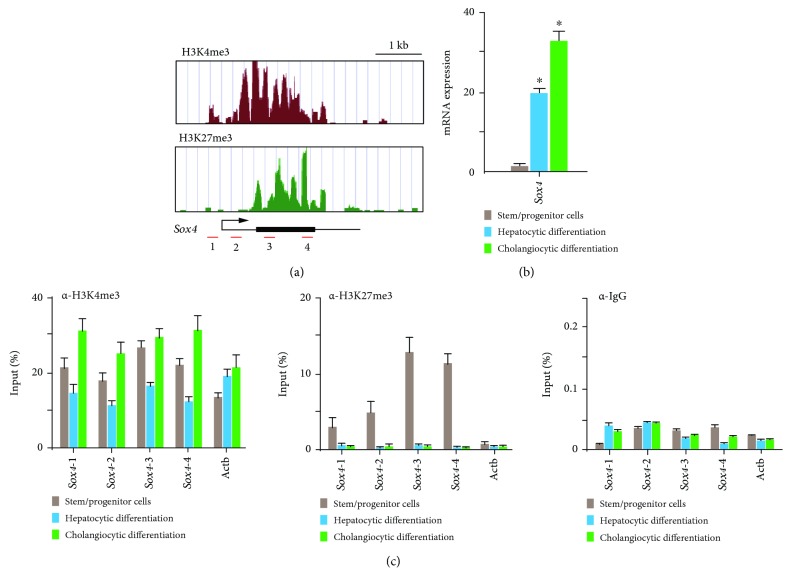
Histone modification status at the *Sox4* locus in purified Dlk^+^ cells and terminally differentiated cells. (a) Signal map of H3K4me3 and H3K27me3 at the Sox4 locus in Dlk^+^ cells. (b) Real-time RT-PCR analysis of *Sox4* in Dlk^+^ cells and cells differentiated toward hepatocyte and cholangiocyte lineages. ^∗^Statistically significant (*P* < 0.05). (c) Quantitative ChIP analyses of the *Sox4* locus and *Actb* control promoter region using anti-H3K4me3 and anti-H3K27me3 antibodies. Percentages of input DNA are shown as mean values for independent triplicate analyses.

Similar to *Ink4a*/*Arf*, *SRY-related HMG box 4* (*Sox4*) exhibited bivalent loci in purified Dlk^+^ cells (Figure 3(a)). Real-time PCR analyses demonstrated that *Sox4* was significantly upregulated after differentiation toward the hepatocyte and cholangiocyte lineages (Figure 3(b)). *Sox4* upregulation was more prominent in cholangiocytes than in hepatocytes. We next focused on the role of *Sox4* in hepatic stem/progenitor cells. *Sox4* upregulation was more prominent during cholangiocytic differentiation than during hepatocytic differentiation. Although high levels of both H3K4me3 and H3K27me3 were detected at the *Sox4* promoter in purified Dlk^+^ cells, only H3K4me3 levels were increased at the *Sox4* locus in terminally differentiated cells (Figure 3(c)). These findings indicate that the *Sox4* expression level is tightly regulated through histone modifications in both stem/progenitor and terminally differentiated cells.

### 3.6. Loss-of-Function Assays of *Sox4* in Hepatic Stem/Progenitor Cells

**Figure 4:**
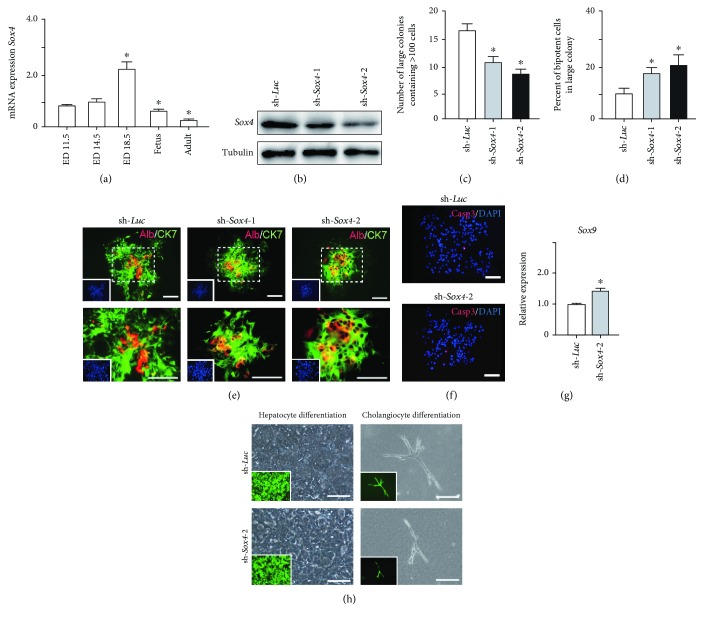
Loss-of-function assays of *Sox4* in Dlk^+^ cells. (a) Real-time RT-PCR analysis of *Sox4* in the fetal liver (ED 11.5, ED14.5, and ED17.5), neonatal liver, and adult liver. ^∗^Statistically significant (*P* < 0.05). (b) Western blot analyses in *Sox4*-knockdown cells using anti-Sox4 and anti-tubulin (loading control) antibodies. (c) The number of large colonies containing >100 cells at day 5. ^∗^Statistically significant (*P* < 0.05). (d) The percentages of Alb^+^CK7^+^ bipotent cells in large colonies at day 5 of culture are shown as mean values for 10 colonies. ^∗^Statistically significant (*P* < 0.05). (e) Fluorescence micrographs of large colonies (containing >100 cells) transduced with indicated viruses at day 5 of culture. Dual immunostaining was performed to detect Alb (red) and CK7 (green) expression. Nuclear DAPI staining (blue) is shown in the insets. Scale bar = 200 *μ*m. (f) Immunostaining analyses demonstrated Casp3 (red) and nuclear DAPI (blue). Scale bar = 200 *μ*m. (g) Real-time RT-PCR analysis of *Sox9* in Dlk^+^ cell-derived colonies. ^∗^Statistically significant (*P* < 0.05). (h) Bright-field images and fluorescence micrographs (inset panels) of cells in EHS gel culture for hepatocytic differentiation and collagen gel culture for cholangiocytic differentiation. Scale bar = 100 *μ*m.

Quantitation of *Sox4* mRNA extracted from fetal, neonatal, and adult livers using real-time RT-PCR demonstrated that the *Sox4* expression achieved a peak in late-gestational fetal liver and decreased rapidly after that (Figure 4(a)). These findings indicated the possibility that derepression of *Sox4* was essential for the normal differentiating process. These results prompted us to test the effects of *Sox4* in a loss-of-function assay. *Sox4* knockdown was confirmed using Western blotting (Figure 4(b)). *Sox4* knockdown impaired colony formation and reduced both the number of large colonies containing >100 cells and the size of colonies (Figure 4(c)). Immunocytochemical analyses revealed an increase in the number of Alb^+^CK7^+^ bipotent cells in colonies derived from *Sox4*-depleted Dlk^+^ cells compared with the findings in control colonies on day 5 of culturing (Figures 4(d) and 4(e)). However, no remarkable differences were observed in a number of Casp3-positive cells in control and *Sox4*-knockdown colonies (Figure 4(f)). In addition, *Sox4* knockdown resulted in a modest but significant increase in *Sox9* mRNA expression (Figure 4(g)). Taken together, *Sox4* is essential for the differentiation of Dlk^+^ cells into mature hepatocytes and cholangiocytes.

To selectively induce terminal hepatocyte maturation, we cultured Dlk^+^ cells in EHS gel supplemented with OSM. Multiple cell clusters with tight cell-cell contact were formed by day 4 of culturing. Compared with the findings for the control clusters, the *Sox4*-knockdown clusters lacked hepatocytes with highly condensed, granulated cytosol and clear round nuclei (Figure 4(h)). Concordant with these findings, real-time RT-PCR analyses revealed that hepatocyte-lineage markers, such as *Alb*, *tyrosine aminotransferase* (*Tat*), *hepatocyte nuclear factor 3β* (*Hnf3β*), and *Hnf4α*, were significantly downregulated in *Sox4*-depleted cells (Supplementary Figure S3(a)). In contrast, stem cell markers, such as *alpha-fetoprotein* (*Afp*), *epithelial cell adhesion molecule* (*Epcam*), and *Bmi1*, were significantly upregulated in *Sox4*-knockdown cells. Next, Dlk^+^ cells were cultured on collagen type I gel in the presence of TNF-*α* to selectively induce cholangiocytic differentiation. *Sox4*-depleted Dlk^+^ cells, but not control Dlk^+^ cells, failed to form tube-like structures (Figure 4(e)). As expected, real-time RT-PCR analyses demonstrated that cholangiocyte-lineage markers, such as *CK7*, *integrin b4* (*Itgb4*), *Hnf1β*, and *Hnf6*, were significantly downregulated, and stem cell markers mentioned above were upregulated in *Sox4*-depleted cells (Supplementary Figure S3(b)). Taken together, Sox4 gene activation is essential for terminal differentiation toward the hepatocyte and cholangiocyte lineages.

## 4. Discussion

Bivalent domains simultaneously contain the opposing histone modification H3K4me3 and H3K27me3 and have been found in the promoters of a broad range of genes that regulate differentiation in ES cells [19]. This elegant mechanism maintains the cell's undifferentiated state while permitting target gene expression to be rapidly activated or repressed when cell fate is determined by differentiation signals [20]. Bivalent domains are intimately involved in the balance between TrxG and PcG histone modifications. In fact, ES cells lacking components of the PcG complex reportedly lose the H3K27me3 histone modification, leading to derepression of bivalent genes [21, 22]. Hence, the undifferentiated state of ES cells is lost, and differentiation accelerates toward a certain cell lineage. Deficits in pluripotency and abnormal differentiation have also been reported in tissue-specific stem cells lacking *Ezh2* [23, 24]. We previously reported that depleting *Ezh2* in hepatic stem/progenitor cells eliminates their capacity of self-renewal and causes them to exhibit abnormal differentiation that is skewed toward the hepatocyte lineage [13]. This suggests that PcG plays an important role in repressing differentiation programs in stem/progenitor cells.

Recently, the popularization of the ChIP-seq analysis, which combines next-generation sequencers with ChIP, has facilitated comprehensive analyses to determine binding sites for transcription factors or enrichments for specific histone modifications [25]. However, few reports have comprehensively analyzed histone modifications in stem/progenitor cells. In the current study, we first performed ChIP-seq analyses using purified Dlk^+^ cells and antibodies against H3K4me3 and H3K27me3. After exploring conditions in which H3K4me3 levels best correlate with gene expression and H3K27me3 levels best inverse-correlate with gene expression, we decided to analyze genes with enrichment of >2-fold (log2) of input in the region within 2.0 kb of the TSSs. We subsequently identified 562 bivalent genes. Of these genes, 26 overlapped with bivalent genes in ES cells, 11 overlapped with bivalent genes in hematopoietic stem cells, and 3 were common to both. Differentiation of hepatic stem/progenitor cells appears to be controlled precisely through epigenetic regulations of bivalent genes. Although these genes are mainly specifically found in hepatic stem/progenitor cells, some of them are commonly found in different stem cell systems.

Next, we integrated ChIP-seq and microarray data to identify bivalent genes that are derepressed when differentiation is induced. Our results indicated that 72 genes were commonly upregulated during differentiation toward both the hepatocyte and cholangiocyte lineages. In fact, one of the genes was *Ink4a*/*Arf*, which is an important PcG target gene. We previously reported that derepression of the *Ink4a*/*Arf* locus in *Bmi1* ^−/−^ Dlk^+^ cells leads to impaired growth activity and self-renewal [18]. Conversely, *Ink4a*/*Arf* ^−/−^ Dlk^+^ cells exhibit remarkable stem/progenitor cell expansion. Further analyses are needed to examine whether 72 genes cited in this study are repressed to maintain pluripotency in hepatic stem/progenitor cells and transcriptionally activated via loss of repressive histone modifications for normal differentiation to proceed.

The 72 aforementioned genes include *Sox4* and *Sox11*. Sox genes are transcription factors belonging to the HMG box superfamily. Some Sox genes are conserved across species, ranging from zebrafish to humans [26]. As their name suggests, *Sox* genes display homology to the DNA-binding HMG box domain of the sex-determination gene *Sry* (generally greater than 50% homology within the HMG box), and to date, more than 20 different *Sox* genes have been cloned from mice [27]. Sox4 plays an important role in regulating differentiation in neural stem and hematopoietic stem/progenitor cells; however, its function in hepatic stem/progenitor cells is unclear [28, 29]. Therefore, we performed a more detailed analysis of Sox4. Although *Sox4* expression in Dlk^+^ cells was extremely low, levels of the transcriptionally repressive H3K27me3 histone modification decreased as the cells were induced to differentiate into hepatocytes and cholangiocytes, thereby leading to 20- and 33-fold increases in *Sox4* expression, respectively. Lentiviral-knockdown of *Sox4* in Dlk^+^ cells impaired terminal differentiation toward hepatocyte and cholangiocyte lineages and causes an increase in Alb^+^CK7^+^ progenitor cells in Dlk^+^ cell-derived colonies. Concordant with these findings, Poncy and colleagues reported that cholangiocyte differentiation and bile duct formation are perturbed in developing livers in *Sox4*-knockout mice [30]. In the present study, *Sox4* knockdown resulted in modest but significant upregulation of *Sox9* expression in Dlk^+^ cell-derived colonies. Given that Sox4 and Sox9 cooperatively regulate development of bile ducts [30], this change might be a compensatory increase. Further analyses are necessary to elucidate the role of Sox4 and interaction between Sox4 and Sox9 in the regulation of hepatic stem/progenitor cells.

Furuyama and colleagues reported that based on a genetic lineage tracing analysis in mice, hepatocytes and cholangiocytes differentiate from *Sox9*-negative hepatoblasts [31]. In the current study, the H3K27me3 modification at the *Sox9* locus was slightly below the cutoff, and it was therefore not included as a bivalent gene. However, *Sox9* expression in Dlk^+^ cells was extremely low, and we observed a greater than 5-fold increase in its expression after the induction of differentiation into hepatocytes or cholangiocytes. We previously revealed that *Sox17* is a target of Bmi1 and that its overexpression in Dlk^+^ cells accelerates their differentiation [18]. These examples illustrate that Sox genes in Dlk^+^ cells are finely controlled at the transcriptional level via histone modifications and suggest that they play important roles in controlling the differentiation of hepatic stem/progenitor cells.

Finally, our findings demonstrated that several genes possess bivalent histone marks in hepatic stem/progenitor cells. Additionally, drastic changes in the expression of these genes caused by the loss of bivalent domains are closely associated with the normal differentiation process. Further analyses are necessary to determine the roles of the identified genes in hepatic stem cell systems.

## Data Availability

The microarray and ChIP-seq data obtained in this study have been deposited in Gene Expression Omnibus (GEO, accession number: GSE 114833) and in the DNA Data Bank of Japan (DDBJ, accession number: DRA006858), respectively. Other data used to support the findings of this study are available from the corresponding author upon request.

## Supplementary Materials

Supplementary Materials Supplementary Figure S1: purification of hepatic stem/progenitor cells. Representative flow cytometric profiles of MACS-sorted Dlk^+^ cells. The percentages of Dlk^+^ cells are indicated as mean values for three independent analyses. Supplementary Figure S2: histone modification status at the *Ink4a/Arf* loci in purified Dlk^+^ and terminally differentiated cells. (a) The signal map of H3K4me3 and H3K27me3 at the Ink4a/Arf locus in Dlk^+^ cells. (b) Real-time RT-PCR analysis of *Ink4a/Arf* in Dlk^+^ cells and cells differentiated toward hepatocyte lineage and cholangiocyte lineages. ^∗^Statistically significant (*P* < 0.05). (c) Quantitative ChIP analyses on the *Ink4a/Arf* loci and Actb control promoter region using anti-H3K4me3 and anti-H3K27me3 antibodies. Percentages of input DNA are shown as mean values for independent triplicate analyses. Supplementary Figure S3: effects of *Sox4*-knockdown on the differentiation of hepatic stem/progenitor cells. (a) Real-time RT-PCR analyses of hepatocytic differentiation and maturation marker genes and stem cell marker genes in EHS gel culture for hepatocytic differentiation. ^∗^Statistically significant (*P* < 0.05). (b) Real-time RT-PCR analyses of cholangiocytic differentiation and maturation marker genes and stem cell marker genes in collagen gel culture for cholangiocytic differentiation. ^∗^Statistically significant (*P* < 0.05). Supplementary Table S1: primer sequences for quantitative RT-PCR. Supplementary Table S2: primer sequences designed for ChIP quantitative PCR. Supplementary Table 3: list of bivalent genes showing upregulation after differentiation induction.
