# Supplementary material for: Genome-Wide Mapping of Bivalent Histone Modifications in Hepatic Stem/Progenitor Cells
Source: Stem Cells Int. 2019 Apr 1;2019:9789240. doi: 10.1155/2019/9789240 (PMC6466853; doi:10.1155/2019/9789240)
Supplement: Supplementary Materials — Supplementary Figure S1: purification of hepatic stem/progenitor cells. Representative flow cytometric profiles of MACS-sorted Dlk+ cells. The percentages of Dlk+ cells are indicated as mean values for three independent analyses. Supplementary Figure S2: histone modification status at the Ink4a/Arf loci in purified Dlk+ and terminally differentiated cells. (a) The signal map of H3K4me3 and H3K27me3 at the Ink4a/Arf locus in Dlk+ cells. (b) Real-time RT-PCR analysis of Ink4a/Arf in Dlk+ cells and cells differentiated toward hepatocyte lineage and cholangiocyte lineages. ∗Statistically significant (P < 0.05). (c) Quantitative ChIP analyses on the Ink4a/Arf loci and Actb control promoter region using anti-H3K4me3 and anti-H3K27me3 antibodies. Percentages of input DNA are shown as mean values for independent triplicate analyses. Supplementary Figure S3: effects of Sox4-knockdown on the differentiation of hepatic stem/progenitor cells. (a) Real-time RT-PCR analyses of hepatocytic differentiation and maturation marker genes and stem cell marker genes in EHS gel culture for hepatocytic differentiation. ∗Statistically significant (P < 0.05). (b) Real-time RT-PCR analyses of cholangiocytic differentiation and maturation marker genes and stem cell marker genes in collagen gel culture for cholangiocytic differentiation. ∗Statistically significant (P < 0.05). Supplementary Table S1: primer sequences for quantitative RT-PCR. Supplementary Table S2: primer sequences designed for ChIP quantitative PCR. Supplementary Table 3: list of bivalent genes showing upregulation after differentiation induction. [file 9789240.f1.pdf]

Supplementary Figure Legends

Supplementary Figure S1 Purification of hepatic stem/progenitor cells. Representative flow cytometric profiles of MACS-sorted Dlk^+ cells. The percentages of Dlk^+ cells are indicated as mean values for three independent analyses.

Supplementary Figure S2 Histone modification status at the *Ink4a*/*Arf* loci in purified Dlk^+ and terminally differentiated cells.

(a) The signal map of H3K4me3 and H3K27me3 at the *Ink4a/Arf* locus in Dlk^+ cells. (b) Real-time RT-PCR analysis of *Ink4a/Arf* in Dlk^+ cells and cells differentiated toward hepatocye lineage and cholangiocyte lineages. *Statistically significant (p <0.05). (c) Quantitative ChIP analyses on the *Ink4a/Arf* loci and *Actb* control promoter region using anti-H3K4me3 and anti-H3K27me3 antibodies. Percentages of input DNA are shown as mean values for independent triplicate analyses.

Supplementary Figure S3 Effects of *Sox4*-knockdown on the differentiation of hepatic stem/progenitor cells.

(a) Real-time RT-PCR analyses of hepatocytic differentiation and maturation marker genes and stem cell marker genes in EHS gel culture for hepatocytic differentiation. *Statistically significant (p <0.05). (b) Real-time RT-PCR analyses of cholangiocytic differentiation and maturation marker genes and stem cell marker genes in collagen gel culture for cholangiocytic differentiation. *Statistically significant (p <0.05).

Supplementary Table S1 Primer sequences for Quantitative RT-PCR

Gene name	Forward primer (5'-3’)	Reverse primer (5'-3’)
p16^{Ink4a}	AATCTCCGCGAGGAAAGC	GTCTGTCTGCAGCGGACTC
p19^{Arf}	GGGTTTTCTTGGTGAAGTTCG	TTGCCCATCATCATCACCT
Hnf1β	CTACGACCGGCAAAAGAATC	CCCCTCGTTGCAAACATT
Hnf3β	CAACCACCCCTTCTCTATCAA	CCTTGAGGTCCATTTTGTGG
Hnf4α	GCGGAGGTCAAGCTACGA	ATCCCAGAGATGGGAGAGGT
Hnf6	AGACCTTCCGGAGGATGTG	TTGCTCTTTCCGTTTGCAG
Alb	TGGAATGCGCAGATGACA	GTGGTTTATCGCAGCAAGTCT
CK7	CATTGAGATCGCCACCTACC	ACAGGTCCCATTCCGTCTC
Itgb4	GAGGATTCATCCAACATCGTG	CGGATGTCCAGGTTGGAG
Tat	CGGAGCAGTCTGTCCACTG	GAACTCCTGGATCCGGCTA
Sox4	CTCGCTCTCCTCGTCCTCT	CGTCTTCGAACTCGTCGTC
Sox9	GTACCCGCATCTGCACAAC	CTCCTCCACGAAGGGTCTCT
Afp	TGGATGTCAGGACAATCTGG	GCAGCTTTGCTTGGACAGT
Epcam	CAGCAAAATATGAGAAGGCTGA	CCAGGTTTATACATCTGCAGTCC
Bmi1	AAACCAGACCACTCCTGAACA	TCTTCTTCTCTTCATCTCATTTTTG
Hprt	TCCTCCTCAGACCGCTTTT	CCTGGTTCATCATCGCTAATC

Supplementary Table S2 Primer sequences designed for ChIP quantitative PCR

Region	Sequence	
Sox4-1	Fw	5'- TTTGCCTCTGTGTGTGTGTG -3'
	Rv	5'- CCTCCAAGCCCCTTGTTATC -3'
Sox4-2	Fw	5'- AATTGCACCAACTCCTCAGC -3'
	Rv	5'- TCGATTGCAGTTCACGAGAG -3'
Sox4-3	Fw	5'- ACAGCGACAAGATTCCGTTC -3'
	Rv	5'- GCCCGACTTCACCTTCTTTC -3'
Sox4-4	Fw	5'- CTCTCCTCGTCCTCTTCCTC -3'
	Rv	5'- CAAAGTTTGAGCTGGGGTTC -3'
Ink4a/Arf-1	Fw	5'- CTTAGAGTTACAGAAAGGGCTGGA -3'
	Rv	5'- GAATTTCAAGGAAGTGCTACCCTA -3'
Ink4a/Arf-2	Fw	5'- CACTGCACTGGAAGAGGACA -3'
	Rv	5'- CTGAAGGTCCTGGGTTCAAA -3'
Ink4a/Arf-3	Fw	5'- GATGGAGCCCGGACTACAGAAG -3'
	Rv	5'- CTGTTTCAACGCCCAGCTCTC -3'
Ink4a/Arf-4	Fw	5'- AGGGAATACACTGTAAGCCTGTGT -3'
	Rv	5'- TTAACTACTCGGATCAGACATCCA -3'
Actb	Fw	5'- CCCAACACACCTAGCAAATTAGAACCAC -3'
	Rv	5'- CCTGGATTGAATGGACAGAGAGTCACT -3'

Supplementary Table 3 List of bivalent genes showing upregulation after differentiation induction

RefSeq	Definition
Adcy1	adenylate cyclase 1
Ap1s2	AP-1 complex subunit sigma-2
Ap3m2	adaptor related protein complex 3 mu 2 subunit
Bmper	BMP binding endothelial regulator
Cbr3	carbonyl reductase 3
Ccdc92	coiled-coil domain containing 92
Cdkn2a	Cyclin dependent kinase inhibitor 2A
Cdr2l	Cerebellar degeneration related protein 2 like
Celsr1	Cadherin EGF LAG seven-pass G-type receptor 1
Crim1	Cysteine rich transmembrane BMP regulator 1
Cyb561	Cytochrome b561
Dlgap4	DLG associated protein 4
Enah	ENAH, actin regulator
Eomes	Eomesodermin
Eps8	Epidermal growth factor receptor pathway substrate 8
Fam171b	Family with sequence similarity 171 member B
Fam189a2	Family with sequence similarity 189 member A2
Fam84a	Family with sequence similarity 84 member A
Fbxo2	F-box protein 2
Fibcd1	Fibrinogen C domain containing 1
Flnc	Filamin C
Fzd9	Frizzled class receptor 9
Gabbr1	Gamma-aminobutyric acid type B receptor subunit 1
Galnt12	Polypeptide N-acetylgalactosaminyltransferase 12
Grasp	General receptor for phosphoinositides 1 associated scaffold pı
Hap1	Huntingtin associated protein 1
Hs3st6	Heparan sulfate-glucosamine 3-sulfotransferase 6
Htra1	HtrA serine peptidase 1
Insm1	INSM transcriptional repressor 1
Irx3	Iroquois homeobox 3
Kcnj4	Potassium voltage-gated channel subfamily J member 4
Kcns3	Potassium voltage-gated channel modifier subfamily S membe
Kif5c	Kinesin family member 5C
Klf5	Kruppel like factor 5
Loxl1	Lysyl oxidase like 1
Mal	Mal, T cell differentiation protein

Supplementary Table 3 Continued

RefSeq	Definition
Mapk12	Mitogen-activated protein kinase 12
Mfsd2a	Major facilitator superfamily domain containing 2A
Myo5a	Myosin VA
Mypop	Myb related transcription factor, partner of profilin
Pfkp	Phosphofructokinase, platelet
Pmp22	Peripheral myelin protein 22
Podxl	Podocalyxin like
Ppm1j	Protein phosphatase, Mg2+/Mn2+ dependent 1J
Prkg2	Protein kinase, cGMP-dependent, type II
Rgl3	Ral guanine nucleotide dissociation stimulator like 3
Rhpn1	Rhophilin Rho GTPase binding protein 1
Rnf113a1	Ring finger protein 113A1
S100a10	S100 calcium binding protein A10
Scn1b	Sodium voltage-gated channel beta subunit 1
Sema7a	Semaphorin 7A
Sfn	Stratifin
Sh2d5	SH2 domain containing 5
Shc2	SHC adaptor protein 2
Slc41a2	Solute carrier family 41 member 2
Sox4	SRY-box 4
Sox11	SRY-box 11
Sp8	Sp8 transcription factor
Suv39h2	Suppressor of variegation 3-9 homolog 2
Thbd	Thrombomodulin
Tmsb10	Thymosin beta 10
Tnfrsf10b	TNF receptor superfamily member 10b
Tpbg	Trophoblast glycoprotein
Tspan2	Tetraspanin 2
Ttc9	Tetratricopeptide repeat domain 9
Vgf	VGF nerve growth factor inducible
Wnt9a	Wnt family member 9A
Wt1	Wilms tumor 1
Zfp462	Zinc finger protein 462
3110043O21Rik	
4930506M07Rik	
E130012A19Rik	

Kanayama et al. Supplementary Figure S1

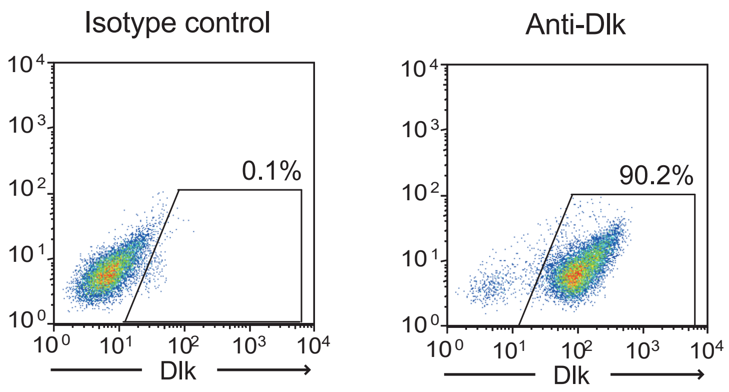

Kanayama et al. Supplementary Figure S2

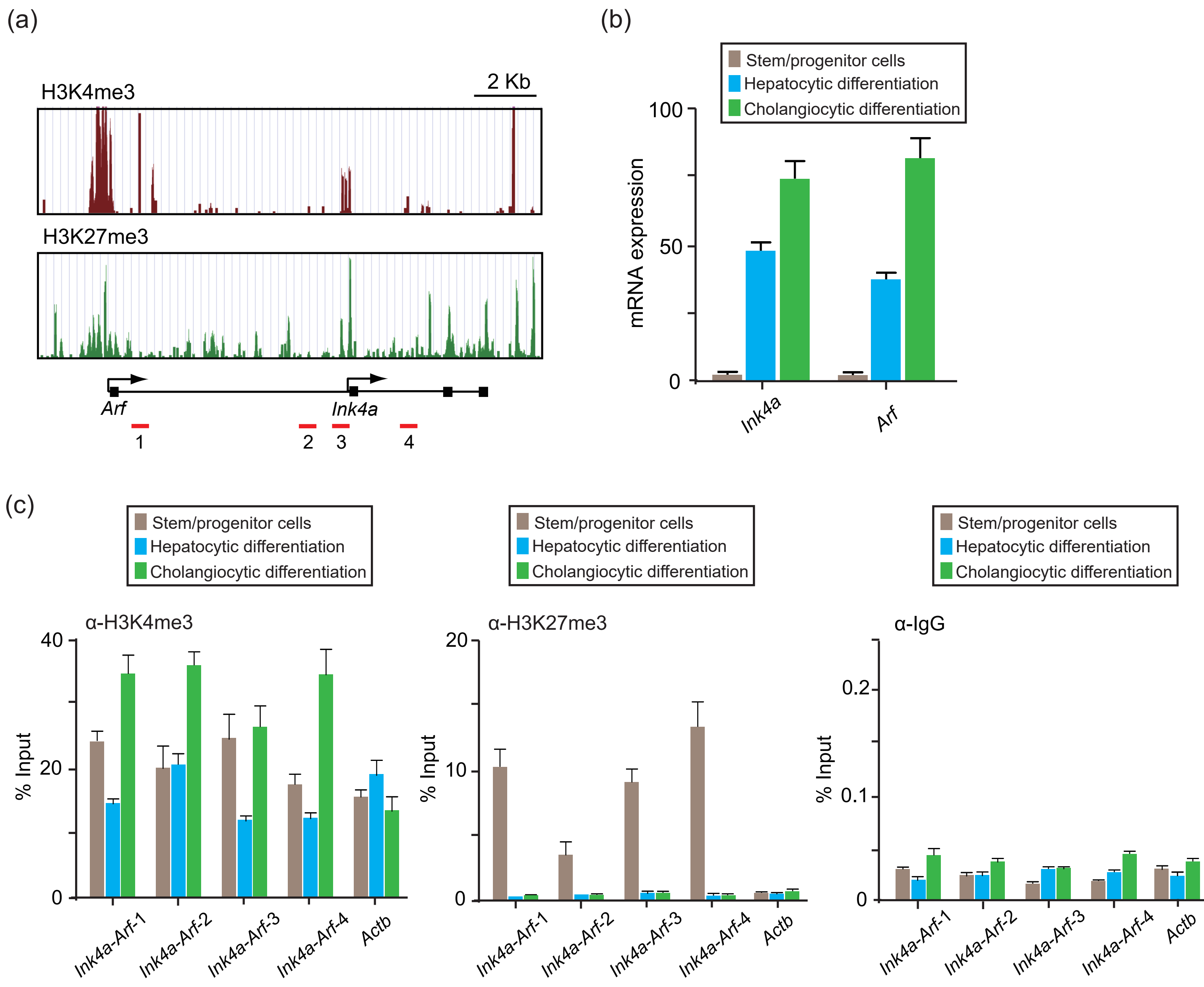

(a)

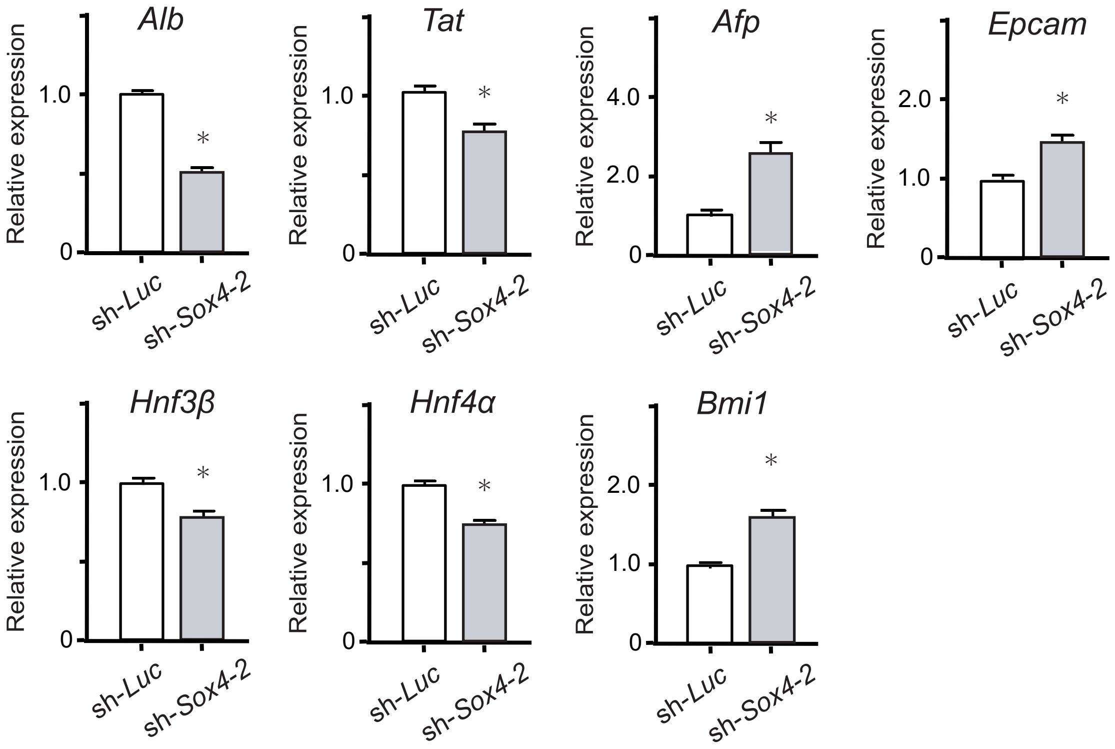
Alb
Tat
Afp
Epcam
Hnf3β
Hnf4α
Bmi1
Relative expression
sh-Luc
sh-Sox4-2

(b)

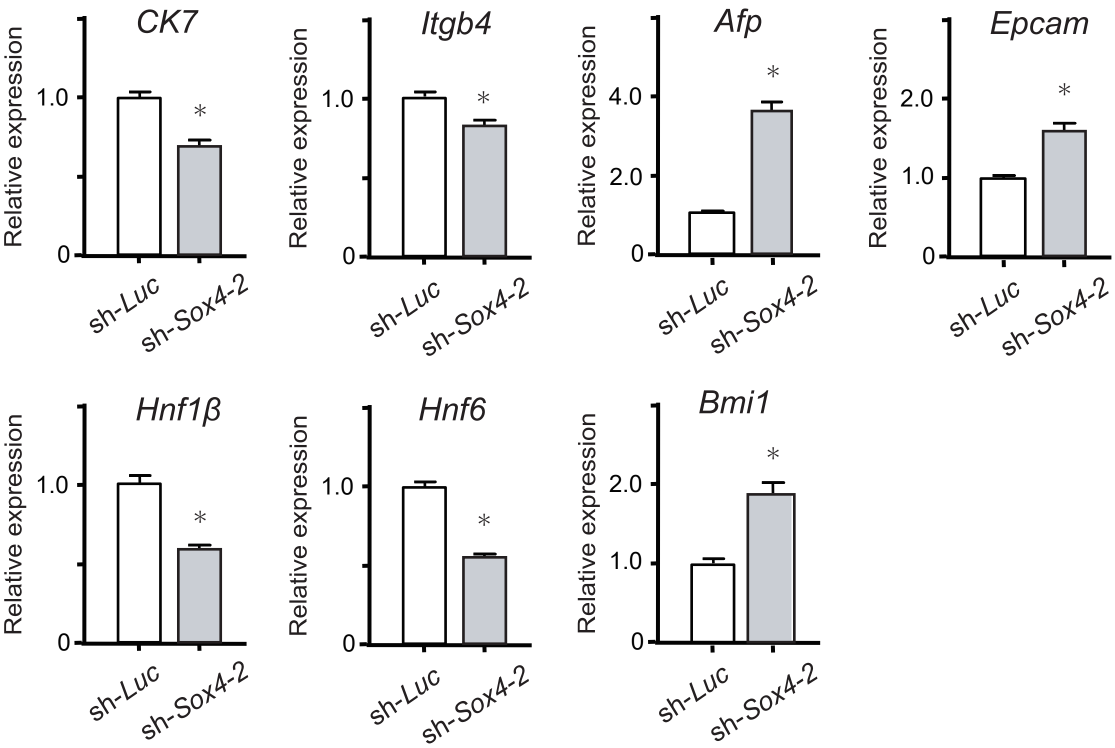
CK7
Itgb4
Afp
Epcam
Hnf1β
Hnf6
Bmi1
Relative expression
sh-Luc
sh-Sox4-2
